# Comparative clinical efficacy of acupuncture combined with manipulation and other non-pharmacological interventions in the treatment of lumbar disc herniation: a prospective, multi-arm, randomized, open-label, blinded endpoint trial

**DOI:** 10.3389/fmed.2024.1507115

**Published:** 2025-01-03

**Authors:** Fudong Shi, Haibao Wen, Yuzhang Liu, Zuoxu Li, Jiao Jin, Ning Liu, Guojun Wang, Chun Chen, Yadi Feng, Hai Lin, Shimin Zhang

**Affiliations:** Wangjing Hospital, China Academy of Chinese Medical Sciences, Beijing, China

**Keywords:** non-pharmacological interventions, acupuncture, manipulation, randomized controlled, clinical trials

## Abstract

**Objective:**

To compare the clinical efficacy and safety of four intervention methods—traditional Chinese manipulation combined with acupuncture, acupuncture alone, manipulation alone, and traction—for the treatment of lumbar disc herniation (LDH).

**Methods:**

A prospective, multi-arm, randomized, parallel-controlled clinical trial was conducted between July 2021 and June 2024. A total of 240 eligible LDH patients were randomized into four groups (60 patients per group) in a 1:1:1:1 ratio: manipulation combined with acupuncture group, manipulation group, acupuncture group, and traction group. Each treatment lasted for 3 weeks. Changes in Visual Analog Scale (VAS) and Japanese Orthopedic Association (JOA) scores were recorded before treatment, at 1 and 3 weeks during treatment, and at 1 and 3 months post-treatment. Adverse events were also monitored.

**Results:**

A total of 210 patients completed the follow-up. At the 3-week (day 21) and 3-month (day 111) follow-ups, the acupuncture + manipulation group showed the most significant improvements, with VAS scores decreasing by 63.34% and 68.30% and JOA scores increasing by 55.17% and 58.33%. The acupuncture group showed VAS score reductions of 55.04% and 59.29% and JOA score increases of 44.52% and 48.29%. The manipulation group reported VAS score reductions of 51.73% and 55.02% and JOA score increases of 41.16% and 45.27%. The traction group demonstrated the least improvement, with VAS scores decreasing by 43.25% and 45.73% and JOA scores increasing by 30.55% and 33.97%. Statistical analysis indicated that the acupuncture + manipulation group had significantly better improvements in VAS and JOA scores than the other three groups during treatment and follow-up periods (*P* < 0.05). There were no significant differences between the acupuncture and manipulation groups (*P* > 0.05), while the traction group showed significantly less improvement compared to the other groups (*P* < 0.05).

**Conclusion:**

This study demonstrates that acupuncture combined with spinal manipulation significantly reduces pain and improves lumbar function in LDH patients compared to other tested interventions. The symptom relief rate was significantly higher in the acupuncture + manipulation group compared to the acupuncture, manipulation, and traction groups.

**Clinical trial registration:**

https://www.chictr.org.cn/index.aspx, identifier ChiCTR2200058598.

## Graphical Abstract

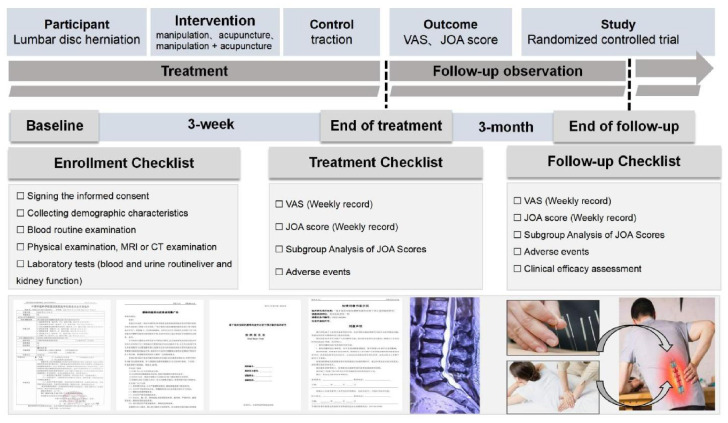

## 1 Introduction

Lumbar disc herniation (LDH) is a common spinal disorder occurring in the lumbar region, primarily affecting working adults aged 30–50 years (1). The intervertebral disc is a soft, resilient substance located between the vertebrae. When the outer fibrous ring of the disc is damaged or ruptured, the inner soft nucleus pulposus may compress surrounding neural structures, leading to the development of LDH (2). The most common symptom is lower back pain, paresthesia, numbness, and tingling which may radiate to the buttocks, thighs, or lower limbs, and occasionally manifesting as atypical symptoms like testicular pain and chronic orchialgia (3) This pain can affect the normal range of motion of the lumbar spine, resulting in stiffness or restricted mobility. Prolonged pain can compress nerve roots, leading to muscle weakness or atrophy. Early diagnosis and treatment are crucial for LDH patients to alleviate pain and restore function. Most patients can achieve relief through non-surgical measures, with only 10%–20% requiring surgical intervention. However, even after surgery, some patients may suffer from residual pain, functional impairments, and psychological issues (4, 5). Additionally, compared to conservative treatments, the benefits of pain relief or functional improvement 1 year after surgery are minimal (6, 7). Although pharmacological treatments are widely used for LDH, they can lead to adverse effects such as nausea, dizziness, fatigue, and mild headaches (8). This is particularly problematic for LDH patients who are pregnant or have gastrointestinal disorders, as they are often more reluctant to take oral painkillers for pain relief (9). Based on current research evidence, non-pharmacological interventions have significant potential to enhance pain management quality in this population. Current guidelines and reviews are increasingly focusing on non-pharmacological treatments for LDH, including exercise, spinal manipulation, and acupuncture (10, 11).

Acupuncture, recognized internationally as a complementary and alternative therapy, is widely recommended as an effective alternative for pain control (12). According to traditional Chinese meridian theory, thin and firm metal needles are inserted through the skin at specific acupoints to treat and relieve lower back and leg pain in LDH patients. A systematic review and meta-analysis found that acupuncture can achieve good therapeutic effects in relieving lower back and leg pain in LDH patients (13).

Spinal manipulation is one of the main methods of traditional Chinese medicine for treating LDH. Through the manipulation and rotation of the spine, the degree of nucleus pulposus protrusion and the position of the nerve root are altered, adhesions and nerve root compression are relieved, and the nerve root canal is expanded. Additionally, spinal manipulation can alleviate muscle spasms, promote muscle relaxation, dilate peripheral blood vessels, improve local anemia and hypoxia, eliminate tissue inflammation and edema, and facilitate the repair of lumbar muscles (14–16).

Many studies have demonstrated the clinical efficacy of non-pharmacological therapies for LDH, including acupuncture, manipulation, and traction. However, the research on these non-pharmacological therapies in this patient population has mostly focused on specific treatment methods longitudinally, either used alone or as part of a combined intervention. To date, no clinical trials have conducted a horizontal comparison of traditional physical therapies (i.e., acupuncture combined with manipulation, acupuncture, manipulation, and traction) in treating LDH patients, and the relative advantages and disadvantages of these therapies remain inconclusive. Therefore, the purpose of this study is to compare the effects of four intervention strategies—traditional Chinese manipulation combined with acupuncture, acupuncture alone, manipulation alone, and traction—on pain, lumbar nerve function, and quality of life in LDH patients, further refining the efficacy evaluation of non-pharmacological interventions with traditional Chinese medicine characteristics for LDH.

## 2 Materials and methods

### 2.1 Study design

**FIGURE 1:**
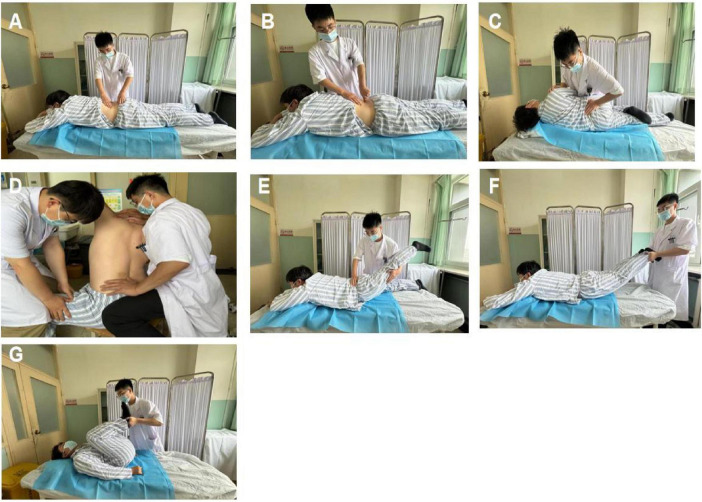
**(A)** Muscle-relaxing waist swaying technique; **(B)** acupressure waist swaying technique; **(C)** lateral rotational bone adjustment technique (lateral or oblique thrust method); **(D)** sitting rotational bone adjustment technique; **(E)** backward leg stretching and thrusting technique; **(F)** prone position traction and shaking technique; **(G)** cross-legged rolling waist technique.

This study was a multi-arm (1:1:1:1 allocation), randomized controlled trial conducted at Wangjing Hospital of China Academy of Chinese Medical Sciences. A total of 240 patients with LDH were recruited and randomly assigned to one of four groups: manipulation combined with acupuncture, manipulation alone, acupuncture alone, or traction. The aim was to evaluate the clinical efficacy of different non-pharmacological interventions in treating LDH. Each group received treatment three times per week for 3 weeks. The primary outcome measures were the Visual Analog Scale (VAS) and the Japanese Orthopedic Association (JOA) scores. The study flowchart is shown in Figure 1.

Each patient underwent a 3-week treatment period followed by a 3-month follow-up. During this time, participants were informed that they could freely withdraw their informed consent at any point during the study.

### 2.2 Trial registration and ethics approval

This study protocol was registered at the Chinese Clinical Trial Registry (registration website: https://www.chictr.org.cn/index.aspx) under the registration number ChiCTR2200058598, and was approved by the Ethics Committee of Wangjing Hospital, China Academy of Chinese Medical Sciences (Approval No.: WJEC-KT-2021-048-P003).

### 2.3 Inclusion criteria and exclusion criteria

The diagnostic criteria for LDH in this study were based on the clinical guidelines of the North American Spine Society (17): (1) history of low back pain; (2) radicular pain in the lower limbs, consistent with the distribution of the nerve roots; (3) sensory abnormalities in the lower limbs, corresponding to the dermatome of the affected nerve; (4) positive straight leg raise test, with aggravated symptoms, and decreased lower limb tendon reflexes; and (5) MRI showing intervertebral disc herniation with nerve compression.

Patients meeting any of the following criteria were included: (1) male or female patients aged 18–60 years; (2) patients with typical clinical symptoms of low back pain, VAS score ≥4, and a confirmed diagnosis of LDH; and (3) patients who were willing to cooperate with treatment and follow-up, and who have signed the informed consent form.

Patients meeting any of the following criteria were excluded from the trial: (1) patients with significant osteophytes, severe lumbar spinal stenosis, spondylolisthesis, or spondylolysis; (2) patients with cauda equina syndrome, conus medullaris syndrome, or other conditions with absolute surgical indications; (3) patients with severe osteoporosis; (4) patients with severe skin lesions, infections, or dermatological conditions at the treatment site; (5) patients with severe cardiovascular, endocrine, or psychiatric disorders; (6) pregnant or lactating patients, or those planning to become pregnant during the study period; and (7) patients with a history of spinal surgery.

### 2.4 Participant recruitment

Participants in this study were recruited through posters at Wangjing Hospital, China Academy of Chinese Medical Sciences. Patients who met the inclusion criteria and provide signed informed consent will enter a screening period. During the screening visit, eligibility was confirmed by experienced clinicians based on patient symptoms, lumbar MRI, or CT images, and a review of each inclusion and exclusion criterion. Patients who met the exclusion criteria were withdrawn from the study within 24 h. Recruitment lasted for 48 months, from July 2022 to June 2024.

### 2.5 Randomization

Eligible participants were randomly assigned in a 1:1:1:1 ratio using a randomization table generated by SAS 9.4 software. The randomization cards were printed and sealed in opaque envelopes. Each of the 240 participants was assigned a sequential number (001-240) according to the order of enrollment. The corresponding numbered envelope was opened, and the treatment group indicated on the card inside was assigned to the participant. Once a random number was assigned, it could not be reassigned. Participants who did not complete the entire study were not replaced.

### 2.6 Blinding

Due to the nature of the four treatment methods, blinding of patients, attending physicians, or researchers was not feasible. To minimize the impact of personal bias on the assessment of study outcomes, the personnel responsible for data collection, endpoint evaluation, and statistical analysis were blinded.

### 2.7 Interventions

During the 3-week treatment period, health education was provided to all enrolled patients, primarily including measures such as wearing a lumbar brace and sufficient bed rest.

#### 2.7.1 Manipulation therapy

(A) Muscle-relaxing waist-shaking method: the patient lies prone. The practitioner places one hand on the patient’s sacrum and uses the other hand to relax and align the paraspinal muscles from top to bottom. Simultaneously, the practitioner rhythmically moves the patient’s waist from side to side. This technique is generally performed for 5–10 min.
(B) Acupressure waist-shaking method: the basic movement is the same as the muscle-relaxing waist-shaking method. While shaking the waist, the thumbs of both hands press along the bladder meridian. This method serves the same purpose as the muscle-relaxing waist-shaking method and can be used alternately.
(C) Lateral rotation manipulation (side or oblique manipulation): the patient lies on their side, with the healthy lower limb naturally extended and the affected limb flexed on top. The practitioner faces the patient and gently applies force in opposite directions on the shoulder and hip, causing the lumbar region to rotate. As resistance is encountered, the force and amplitude of the manipulation are increased. A “cracking” sound is often heard at this point.
(D) Sitting rotation manipulation: The patient sits with the lumbar region relaxed, while an assistant stands beside them, stabilizing one leg with one hand and holding the patient’s shoulder with the other. The practitioner presses against the misaligned spinous process with one hand, and with the other hand, passes under the patient’s armpit to hold the opposite shoulder or neck. This maneuver is performed in three steps. First, the patient is instructed to flex the spine forward. When the spinous process gap under the thumb opens, the position is stabilized. The patient is then asked to rotate maximally to that side. Finally, the practitioner rotates the patient’s lumbar region while the assistant stabilizes the pelvis, often producing a “cracking” sound as the spinous process moves under the practitioner’s thumb.
(E) Backward leg-stretching manipulation: the patient lies prone, elbows bent, with hands under the chin. The practitioner stands to the side, pressing on the lumbar region with one hand to limit lumbar extension while lifting and pulling the lower limb backward with the other hand. The hands coordinate to mobilize the sacroiliac joint.
(F) Prone traction manipulation: the patient lies prone, gripping the head of the bed with both hands. The practitioner holds the patient’s ankles, performing sustained traction while gently shaking the ankles to induce lateral movement of the lumbar region.
(G) Cross-leg rolling manipulation: the patient lies supine with knees and hips flexed. The practitioner holds the patient’s heels, moving the toes toward the ceiling until the legs extend beyond the patient’s chest. This rhythmic movement is repeated harmoniously to enhance spinal flexion, typically lasting for about 2 min.

The treatment duration is 20–30 min.

#### 2.7.2 Acupuncture therapy

(1) Acupoint selection: Bilateral Jiaji (EX-B2), Dachangshu (BL25), Shenshu (BL23), Huantiao (GB30); healthy-side Houxi (SI3), Weizhong (BL40), Chengshan (BL57).
(2) Needles: disposable sterile acupuncture needles (0.25 mm × 40 mm) manufactured by Han Yi, Tianjin, are used for all points except Huantiao, where 0.25 mm × 60 mm needles are used.
(3) Procedure: the patient lies prone, with the lumbar region exposed. After disinfection of the acupoints, bilateral Jiaji, Dachangshu, Shenshu, Huantiao, and healthy-side Houxi, Weizhong, and Chengshan are punctured perpendicularly at a 90 angle using standard reinforcing and reducing methods. Needles are retained for 30 min after achieving a needling sensation or radiating pain. After needle removal, sterile dry cotton swabs are used to stop bleeding. The same physician administers all treatments in each group.

#### 2.7.3 Traction therapy

For the first traction session, patients start with 40% of their body weight, gradually increasing to 50%. For elderly or frail patients, they start with 30% of their body weight, gradually increasing to 40%. The traction weight and duration can be varied, with heavier weights applied for shorter durations and lighter weights for longer durations. Each session lasts 20–30 min, using a Sanyo OL-2000 computer traction bed manufactured by Japanese Okuchi Technology Co., Ltd. All the treatments described above are administered three times a week, on alternate days.

### 2.8 Outcome measures

The primary outcomes were assessed by observing changes in VAS and JOA scores at baseline, 1 week, 3 weeks post-treatment, 1 month follow-up, and 3 months follow-up to evaluate improvements in pain and lumbar nerve function. Secondly, the clinical efficacy of the patients was evaluated at 1 week, 3 weeks post-treatment, 1 month follow-up, and 3 months follow-up using the JOA improvement criteria: improvement rate <25% as poor, 25%–50% as fair, 50%–74% as good, and 75%–100% as excellent.

#### 2.8.1 Safety indicators and treatment of adverse events

An adverse event record was established, and any adverse reactions occurring during the trial were promptly recorded, including the time of onset, severity, duration, and treatment measures. The cause of the adverse event was analyzed, and its relevance to the trial was assessed. If symptoms such as palpitations, dizziness, blurred vision, or excessive sweating occurred after acupuncture, the procedure was immediately stopped, and the patient was placed in a supine position and given warm water. If lumbar or leg pain worsened after manipulation or traction, the intensity and duration of the manipulation or the weight and duration of traction were adjusted according to the patient’s tolerance and individual differences.

During the treatment period, if patients experienced adverse reactions such as worsening low back pain or increased lower limb numbness due to intolerance to the manipulation or traction therapy, the study was temporarily suspended. If the symptoms did not improve, 250 ml of mannitol was administered for dehydration, depending on the patient’s condition. For severe pain, non-steroidal anti-inflammatory drugs (NSAIDs) were prescribed, such as one tablet of loxoprofen sodium orally, three times a day. If the symptoms, such as back and leg pain or numbness, persisted or worsened, the study was discontinued based on the patient’s preference.

### 2.9 Sample size calculation

The sample size calculation was performed using Power and Sample Size (PASS) software (version 11.0). The primary outcome was the mean VAS score after 3 weeks of treatment. Based on our preliminary and literature studies (18), the mean VAS scores after 3 weeks of treatment were 2.73, 3.10, 3.22, and 3.30 for the acupuncture plus manipulation group, acupuncture group, manipulation group, and traction group, respectively, with standard deviations of 0.79, 0.71, 0.45, and 0.67. The two-sided significance level was set at 5% (α), and the power was 90% (β), requiring 48 patients per group. Considering a 20% dropout rate, the sample size for each group was increased to 60 patients, resulting in a total required sample size of 240 patients.

#### 2.9.1 Statistical methods

Statistical analysis was conducted using SPSS version 25.0. All statistical tests were two-sided, with a significance level of α = 0.05, meaning differences were considered statistically significant if *P* < 0.05. Categorical variables were described using counts or percentages and analyzed using the chi-square test (χ^2^ test). Continuous variables were described by calculating the mean, standard deviation, or 95% confidence interval (95% CI). Data were tested for normality and homogeneity of variance. Multiple group comparisons were conducted using one-way analysis of variance (ANOVA), and pairwise comparisons were performed using the least significant difference (LSD)-*t* test; non-parametric rank-sum tests were used when variance was unequal.

## 3 Results

**FIGURE 2:**
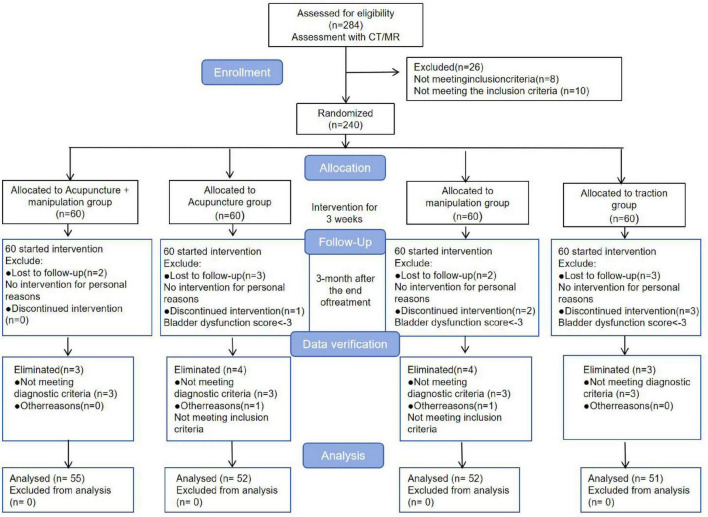
Consolidated Standards of Reporting Trials (CONSORT) flow charts for enrollment and follow-up of all participants throughout the trial period.

Between July 2022 and June 2024, 284 patients were screened for this study, of which 44 were excluded due to lumbar MRI or CT findings or failure to meet inclusion criteria, leaving 240 patients enrolled in the trial. The 240 patients were divided into four groups: acupuncture plus manipulation group, acupuncture group, manipulation group, and traction group, with 60 patients in each group. During follow-up, five patients withdrew from the acupuncture plus manipulation group, eight patients each from the acupuncture and manipulation groups, and nine patients from the traction group. Thus, a total of 210 patients completed the follow-up and were included in the statistical analysis. The screening process of all participants is shown in Figure 2.

### 3.1 Baseline characteristics

The detailed demographic and clinical characteristics of all participants are shown in Table 1. All participants were under the age of 60 years (mean age 41.16 ± 13.52), with the majority being female (57.14%) and of Han ethnicity (94.76%). Most participants had a normal BMI (BMI between 18 and 24), and their body temperature, pulse, and respiratory rate fluctuated within normal ranges. However, the duration of disease and time since onset were prolonged for all participants, with an average of 1,089.66 ± 733.54 days. SD, standard deviation; BMI, body mass index; VAS, Visual Analog Scale (0–10); JOA, Japanese Orthopedic Association scores (3 to 29); SF-36, 36-Item Short Form Health Survey. The duration of disease refers to the course and progression since the diagnosis of LDH, and the onset time refers to the time since the first day of symptom appearance.

**TABLE 1:** Baseline characteristics of the participants.

Characteristic	All participants (*n* = 210)	AM group (*n* = 55)	Ac group (*n* = 52)	SM group (*n* = 52)	Tr group (*n* = 51)	Statistic	*P* value
Age, mean (SD)	44.68 ± 10.73	46.51 ± 10.92	45.10 ± 10.04	42.92 ± 10.99	44.04 ± 10.72	3.60 (*Z*)	0.308
Female, No. (%)	120 (57.14)	32 (26.67)	29 (24.17)	31 (25.83)	28 (23.33)	0.345 (χ^2^)	0.951
Han nationality, No. (%)	199 (94.76)	52 (26.13)	50 (25.13)	48 (24.12)	49 (24.62)	1.017 (χ^2^)	0.797
BMI, mean (SD), kg/m^2^	23.14 ± 4.01	23.34 ± 4.23	23.19 ± 4.11	23.09 ± 3.98	23.39 ± 4.16	6.899 (*Z*)	0.075
Temperature, mean (SD), °C	36.45 ± 0.29	36.25 ± 0.31	36.33 ± 0.34	36.58 ± 0.29	36.33 ± 0.33	1.067 (*Z*)	0.785
Pulse, mean (SD), cpm	74.93 ± 6.14	74.67 ± 6.24	74.56 ± 6.31	74.63 ± 6.55	74.81 ± 6.39	1.509 (*Z*)	0.680
Respiration, mean (SD), cpm	18.38 ± 2.98	18.26 ± 3.02	18.47 ± 2.65	18.41 ± 3.19	18.19 ± 3.08	7.173 (*Z*)	0.067
Course of disease, mean (SD), d	1,089.66 ± 733.54	1,021.31 ± 866.43	1,099.45 ± 977.56	1,021.23 ± 834.23	1,003.23 ± 934.23	0.334 (*Z*)	0.954
Time of onset, mean (SD), d	48.34 ± 39.04	47.25 ± 40.23	46.25 ± 39.89	43.55 ± 36.71	44.65 ± 39.54	4.313 (*Z*)	0.230
VAS score	5.78 ± 0.98	5.83 ± 1.03	5.69 ± 0.78	5.71 ± 1.05	5.66 ± 1.16	0.412 (*F*)	0.794
JOA score	13.23 ± 3.17	12.89 ± 3.51	13.37 ± 2.76	13.26 ± 3.57	13.49 ± 3.90	0.442 (*F*)	0.724

### 3.2 Visual Analog Scale scores

**FIGURE 3:**
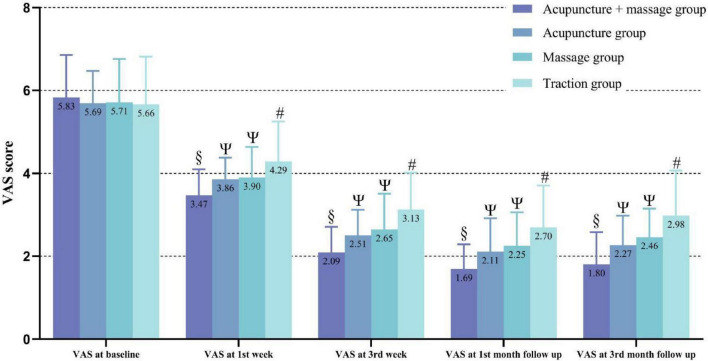
Lower values correspond to clinical improvement. “#” indicates a statistically significant difference compared to pre-treatment within the traction group; “Ψ” indicates that the acupuncture and manipulation groups continued to show significant improvement in VAS scores at all follow-up points, with VAS scores significantly lower than those of the traction group at all follow-up points; “§” indicates that the acupuncture combined with manipulation group continued to show significant improvement in VAS scores at all follow-up points, with VAS scores significantly lower than those of the other groups at all follow-up points.

Visual Analog Scale is the primary indicator for assessing pain improvement. At the start of treatment, there were no statistically significant differences in VAS scores among the four groups (*P* > 0.05). After treatment, all four groups showed a decrease in VAS scores compared to pre-treatment levels, with the acupuncture combined with manipulation group showing a significantly greater reduction in VAS scores than the other three groups (*P* < 0.05). Conversely, the traction group exhibited a significantly smaller reduction in VAS scores compared to the other three groups (*P* < 0.05). It is noteworthy that although the acupuncture group had a greater reduction in VAS scores than the manipulation group, the difference was not statistically significant (*P* > 0.05). According to our research, at the 1-month follow-up, the reductions in VAS scores were greatest in the acupuncture combined with manipulation group, acupuncture group, manipulation group, and traction group, at 70.64% (67.78, 73.50), 62.35% (58.15, 66.55), 58.70% (53.64, 63.76), and 50.39% (44.51, 56.27), respectively. Although the VAS scores increased slightly at the 3-month follow-up compared to the 1-month follow-up, the difference was not statistically significant. Moreover, VAS scores remained lower than pre-treatment levels, without any rebound effect. These changes are illustrated in Table 2 and Figure 3.

**TABLE 2:** Study outcomes across study time points.

Time	AM group (*n* = 55) Mean (95% CI)	Ac group (*n* = 52) Mean (95% CI)	SM group (*n* = 52) Mean (95% CI)	Tr group (*n* = 51) Mean (95% CI)
**Average VAS**				
Baseline	5.83 (5.56–6.12)	5.69 (5.47–5.91)	5.71 (5.42–6.01)	5.66 (5.34–5.99)
1 week	3.47 (3.30–3.64)	3.86 (3.72–4.01)	3.90 (3.70–4.11)	4.29 (4.02–4.57)
3 week	2.09 (1.92–2.26)	2.51 (2.35–2.69)	2.65 (2.41–2.89)	3.13 (2.89–3.39)
1 month follow-up	1.69 (1.53–1.85)	2.11 (1.89–2.34)	2.25 (2.02–2.48)	2.70 (2.42–2.99)
3 month follow-up	1.80 (1.59–2.01)	2.27 (2.07–2.47)	2.46 (2.27–2.66)	2.98 (2.65–3.32)
**VAS DR**				
1 week	38.76% (34.60, 42.93)	31.22% (28.12, 34.33)	29.74% (25.07, 34.40)	22.63% (17.82, 27.44)
3 week	63.34% (60.11, 66.57)	55.04% (51.71, 58.38)	51.73% (46.47, 56.98)	43.25% (38.46, 48.03)
1 month follow-up	70.64% (67.78, 73.50)	62.35% (58.15, 66.55)	58.70% (53.64, 63.76)	50.39% (44.51, 56.27)
3 month follow-up	68.30% (64.25, 72.36)	59.29% (55.52, 63.05)	55.02% (50.32, 59.72)	45.73% (39.25, 52.21)
**Average JOA**				
Baseline	12.89 (11.94–13.83)	13.37 (12.60–14.13)	13.26 (12.28–14.26)	13.49 (12.39–14.59)
1 week	18.60 (18.09–19.10)	17.50 (17.08–17.92)	17.56 (16.99–18.12)	16.45 (15.75–17.15)
3 week	20.58 (20.15–21.02)	19.50 (19.03–19.97)	19.10 (18.48–19.71)	17.86 (17.21–18.51)
1 month follow-up	21.0 (20.55–21.45)	19.92 (19.35–20.50)	19.65 (19.09–20.22)	18.47 (17.77–19.17)
3 month follow-up	20.71 (20.08–21.27)	19.50 (19.01–19.99)	19.04 (18.57–19.50)	17.78 (16.96–18.61)
**JOA IR**				
1 week	28.18% (23.23, 33.13)	15.43% (11.51, 19.34)	14.61% (9.31, 19.91)	6.65% (1.02, 12.27)
3 week	55.17% (51.13, 59.21)	44.52% (40.02, 49.02)	41.16% (34.88, 47.44)	30.55% (24.86, 36.24)
1 month follow-up	61.85% (57.69, 66.02)	49.94% (44.72, 55.16)	49.18% (43.12, 55.25)	39.41% (32.43, 46.39)
3 month follow-up	58.33% (53.29, 63.36)	48.29% (43.58, 53.00)	45.27% (39.42, 51.11)	33.97% (26.2, 41.74)

### 3.3 Japanese Orthopedic Association scores

**FIGURE 4:**
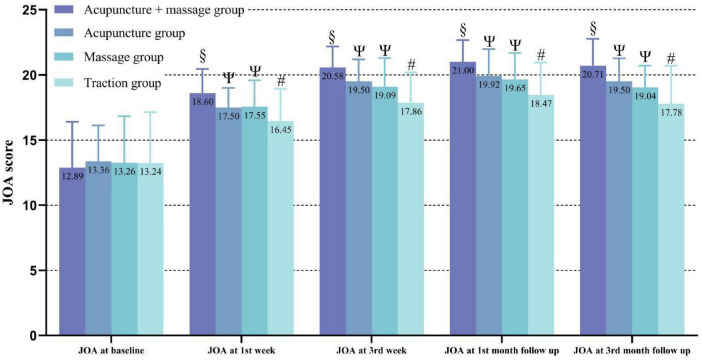
Changes in JOA scores over time among the groups, higher values correspond to clinical improvement. “#” indicates a statistically significant difference compared to pre-treatment within the traction group; “Ψ” indicates that the acupuncture and manipulation groups continued to show significant improvement in JOA scores at all follow-up points, with JOA scores significantly higher than those of the traction group at all follow-up points; “§” indicates that the acupuncture combined with manipulation group continued to show significant improvement in JOA scores at all follow-up points, with JOA scores significantly higher than those of the other groups at all follow-up points.

Japanese Orthopedic Association is a key indicator for assessing symptom improvement and functional recovery in patients with LDH. At the start of treatment, there were no statistically significant differences in JOA scores among the four groups (*P* > 0.05). After treatment, all four groups showed an increase in JOA scores, with the acupuncture combined with manipulation group demonstrating significantly greater improvement in JOA scores compared to the other three groups, while the traction group showed significantly less improvement compared to the other three groups (*P* < 0.05). It is noteworthy that although the acupuncture group had higher JOA scores than the manipulation group, the difference was not statistically significant (*P* > 0.05). The specific changes are illustrated in Table 2 and Figure 4.

#### 3.3.1 Subgroup analysis of JOA scores

Subgroup analysis of JOA scores revealed that the mean values of most indicators in the acupuncture combined with manipulation group were higher than those in the other groups, while the mean values in the traction group were significantly lower than those in the other groups. Additionally, the results showed: (1) At 3 weeks post-treatment and at 1 month follow-up, the acupuncture combined with manipulation group showed statistically significant improvement in daily activities such as back pain, gait, lower limb sensation, washing, bending, and sitting scores, as well as in back pain, washing, and sitting scores at 3 months follow-up, compared to the other three groups (*P* < 0.05). (2) The acupuncture group showed statistically significant improvement in limb numbness scores at 3 weeks post-treatment compared to the manipulation and traction groups (*P* < 0.05). (3) The manipulation group showed statistically significant improvement in back pain scores at 3 weeks post-treatment compared to the acupuncture and traction groups (*P* < 0.05). However, there was no significant difference in lumbar flexion scores between the manipulation and traction groups (*P* > 0.05), but both were significantly better than the acupuncture group (*P* < 0.05). (4) Notably, at the 1-month and 3-month follow-ups, there were no significant differences in the JOA subgroup scores between the acupuncture and manipulation groups (*P* > 0.05). Detailed results are shown in Table 3.

**TABLE 3:** JOA score subgroup analysis across study time.

		AM group (*n* = 55)	Ac group (*n* = 52)	SM group (*n* = 52)	Tr group (*n* = 51)	AM group (*n* = 55)	Ac group (*n* = 52)	SM group (*n* = 52)	Tr group (*n* = 51)
Subjective symptoms (9 points)	Low back pain	1.18 (1.01, 1.29)	1.12 (1.02, 1.27)	1.16 (1.01, 1.25)	1.15 (1.00, 1.21)	1.67 (1.41, 1.79)	1.56 (1.32, 1.27)	1.57 (1.31, 1.65)	1.45 (1.20, 1.61)
	Leg pain and tingling	1.59 (1.42, 1.70)	1.51 (1.41, 1.65)	1.53 (1.43, 1.62)	1.51 (1.41, 1.61)	1.79 (1.42, 1.90)	1.71 (1.43, 1.85)	1.69 (1.45, 1.88)	1.55 (1.42, 1.69)
	Gait	2.19 (2.09, 2.37)	2.11 (1.99, 2.19)	2.15 (1.98, 2.21)	2.11 (1.91, 2.23)	2.31 (2.19, 2.57)	2.12 (1.99, 2.21)	2.14 (1.99, 2.31)	1.99 (1.51, 2.03)
Clinical signs (6 points)	Straight leg raising test	1.19 (1.04, 1.15)	1.09 (0.99, 1.19)	1.11 (0.92, 1.21)	1.12 (0.91, 1.23)	1.23 (1.14, 1.35)	1.19 (0.99, 1.39)	1.16 (1.02, 1.31)	1.15 (0.90, 1.24)
	Sensory disturbance	1.39 (1.32, 1.61)	1.35 (1.22, 1.51)	1.31 (1.13, 1.41)	1.32 (1.11, 1.47)	1.51 (1.34, 1.71)	1.44 (1.32, 1.52)	1.41 (1.23, 1.51)	1.38 (1.21, 1.44)
	Motor disturbance	1.60 (1.49, 1.69)	1.56 (1.47, 1.62)	1.51 (1.34, 1.67)	1.53 (1.31, 1.69)	1.62 (1.39, 1.79)	1.57 (1.37, 1.65)	1.56 (1.36, 1.69)	1.49 (1.21, 1.71)
Restriction of activities of daily living (14 points)	Turning over while lying down	1.21 (1.11, 1.39)	1.15 (1.01, 1.36)	1.17 (1.00, 1.29)	1.19 (1.01, 1.31)	1.39 (1.15, 1.69)	1.31 (1.21, 1.47)	1.25 (1.10, 1.39)	1.21 (1.00, 1.41)
	Standing	1.31 (1.20, 1.41)	1.28 (1.19, 1.37)	1.30 (1.21, 1.42)	1.32 (1.19, 1.40)	1.51 (1.26, 1.65)	1.41 (1.29, 1.56)	1.36 (1.22, 1.46)	1.23 (1.09, 1.42)
	Walking	1.26 (1.19, 1.31)	1.19 (1.13, 1.32)	1.22 (1.11, 1.31)	1.24 (1.09, 1.32)	1.43 (1.18, 1.52)	1.35 (1.23, 1.56)	1.32 (1.16, 1.51)	1.34 (1.07, 1.42)
	Washing face	1.25 (1.15, 1.35)	1.22 (1.12, 1.34)	1.20 (1.09, 1.36)	1.24 (1.02, 1.34)	1.51 (1.43, 1.69)	1.44 (1.13, 1.64)	1.36 (1.19, 1.55)	1.27 (1.01, 1.44)
	Leaning forward	1.16 (1.09, 1.25)	1.09 (1.00, 1.29)	1.17 (1.03, 1.39)	1.16 (1.02, 1.32)	1.46 (1.39, 1.65)	1.31 (1.20, 1.59)	1.41 (1.23, 1.59)	1.40 (1.12, 1.49)
	Sitting	0.98 (0.89, 1.02)	0.91 (0.88, 1.06)	0.97 (0.86, 1.11)	0.92 (0.85, 1.12)	1.21 (0.99, 1.32)	1.13 (1.08, 1.36)	1.01 (0.96, 1.21)	0.98 (0.82, 1.21)
	Lifting or holding	1.01 (0.91, 1.10)	1.03 (0.92, 1.12)	1.09 (0.91, 1.18)	1.02 (0.89, 1.19)	1.24 (1.11, 1.40)	1.04 (0.95, 1.19)	0.99 (0.90, 1.12)	1.12 (0.87, 1.16)
	Biadder function	0 (0, 0)	0 (0, 0)	0 (0, 0)	0 (0, 0)	0 (0, 0)	0 (0, 0)	0 (0, 0)	0 (0, 0)
		**Baseline**				**JOA at 3 week**			
		**AM group** **(*n* = 55)**	**Ac group** **(*n* = 52)**	**SM group** **(*n* = 52)**	**Tr group** **(*n* = 51)**	**AM group** **(*n* = 55)**	**Ac group** **(*n* = 52)**	**SM group** **(*n* = 52)**	**Tr group** **(*n* = 51)**
Subjective symptoms (9 points)	Low back pain	1.89 (1.61, 1.96)	1.69 (1.42, 1.87)	1.66 (1.31, 1.75)	1.50 (1.10, 1.51)	1.85 (1.71, 1.96)	1.67 (1.32, 1.97)	1.56 (1.41, 1.75)	1.35 (1.00, 1.31)
	Leg pain and tingling	2.11 (1.82, 2.30)	1.99 (1.91, 2.15)	1.89 (1.53, 1.92)	1.65 (1.41, 1.71)	2.04 (1.93, 2.25)	1.91 (1.51, 2.15)	1.83 (1.43, 1.92)	1.61 (1.42, 1.81)
	Gait	2.49 (2.29, 2.67)	2.21 (1.95, 2.39)	2.16 (1.98, 2.41)	2.09 (1.94, 2.33)	2.42 (2.11, 2.67)	2.19 (1.99, 2.49)	2.11 (1.98, 2.41)	1.91 (1.61, 2.13)
Clinical signs (6 points)	Straight leg raising test	1.41 (1.24, 1.63)	1.43 (1.29, 1.69)	1.45 (1.22, 1.51)	1.36 (1.11, 1.49)	1.46 (1.34, 1.59)	1.39 (1.19, 1.69)	1.41 (1.22, 1.71)	1.32 (1.11, 1.63)
	Sensory disturbance	1.72 (1.63, 1.86)	1.67 (1.42, 1.81)	1.37 (1.23, 1.51)	1.29 (1.01, 1.39)	1.67 (1.41, 1.81)	1.62 (1.32, 1.91)	1.34 (1.23, 1.51)	1.22 (1.12, 1.47)
	Motor disturbance	1.79 (1.46, 1.91)	1.69 (1.57, 1.82)	1.58 (1.35, 1.77)	1.49 (1.34, 1.66)	1.71 (1.45, 1.99)	1.66 (1.37, 1.83)	1.54 (1.32, 1.77)	1.43 (1.21, 1.62)
Restriction of activities of daily living (14 points)	Turning over while lying down	1.61 (1.41, 1.89)	1.45 (1.31, 1.66)	1.21 (1.10, 1.59)	1.31 (1.03, 1.41)	1.54 (1.21, 1.79)	1.46 (1.21, 1.76)	1.12 (1.02, 1.39)	1.19 (1.02, 1.41)
	Standing	1.62 (1.40, 1.81)	1.58 (1.39, 1.87)	1.38 (1.24, 1.52)	1.29 (1.14, 1.42)	1.52 (1.21, 1.77)	1.48 (1.15, 1.67)	1.32 (1.19, 1.48)	1.22 (1.13, 1.41)
	Walking	1.60 (1.39, 1.78)	1.49 (1.23, 1.82)	1.48 (1.21, 1.61)	1.39 (1.29, 1.52)	1.51 (1.39, 1.81)	1.45 (1.23, 1.72)	1.42 (1.21, 1.71)	1.34 (1.19, 1.58)
	Washing face	1.75 (1.55, 1.95)	1.56 (1.42, 1.64)	1.48 (1.29, 1.66)	1.31 (1.12, 1.35)	1.75 (1.45, 1.95)	1.52 (1.32, 1.74)	1.46 (1.29, 1.66)	1.22 (1.04, 1.64)
	Leaning forward	1.66 (1.59, 1.85)	1.53 (1.30, 1.79)	1.54 (1.33, 1.79)	1.34 (1.22, 1.52)	1.56 (1.39, 1.85)	1.49 (1.23, 1.79)	1.47 (1.13, 1.69)	1.26 (1.12, 1.38)
	Sitting	1.39 (1.29, 1.72)	1.29 (1.18, 1.36)	1.23 (1.06, 1.31)	1.31 (1.05, 1.42)	1.38 (1.19, 1.59)	1.25 (1.08, 1.36)	1.17 (0.96, 1.31)	0.97 (0.81, 1.13)
	Lifting or holding	1.37 (1.21, 1.60)	1.37 (1.12, 1.52)	1.47 (1.21, 1.58)	1.26 (1.02, 1.49)	1.31 (1.21, 1.52)	1.34 (1.12, 1.52)	1.39 (1.19, 1.58)	1.04 (0.85, 1.14)
	Biadder function	0 (0, 0)	0 (0, 0)	0 (0, 0)	0 (0, 0)	0 (0, 0)	0 (0, 0)	0 (0, 0)	0 (0, 0)
		**JOA at l month follow-up**				**JOA at 3 month follow-up**			
								*p* < 0.05	*p* > 0.05

### 3.4 Clinical efficacy assessment

**FIGURE 5:**
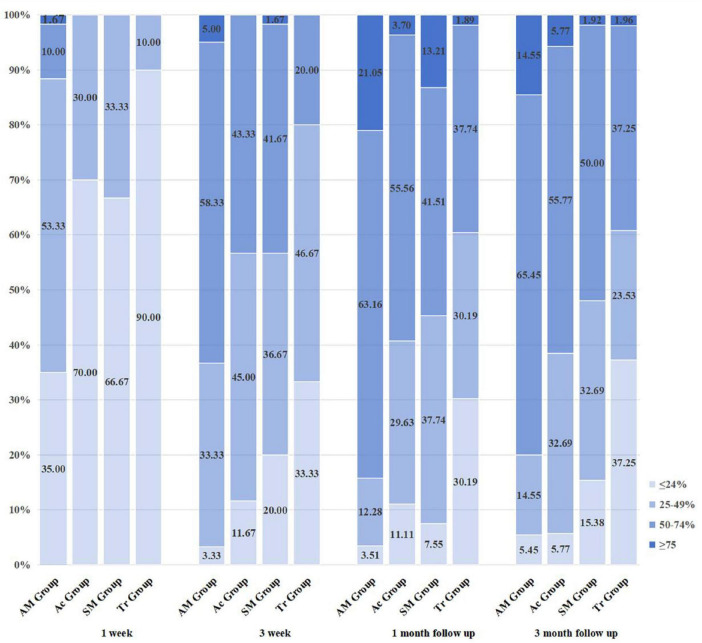
Comparison of clinical efficacy among the four groups using the Kruskal–Wallis.

Based on the efficacy criteria, a comparison among the four groups was conducted. After 1 week of treatment, the rate of symptom and sign improvement in the acupuncture combined with manipulation group was significantly higher than that of the other three groups, with a statistically significant difference (*P* < 0.05). However, there were no significant differences among the acupuncture, manipulation, and traction groups (*P* > 0.05). After 3 weeks of treatment, the acupuncture combined with manipulation group continued to show significantly better improvement than the manipulation and traction groups (*P* < 0.05), while no significant differences were observed among the acupuncture, acupuncture combined with manipulation, and manipulation groups (*P* > 0.05). The acupuncture and manipulation groups showed significantly better improvement than the traction group, with statistical significance (*P* < 0.05). At the 1-month follow-up, the acupuncture combined with manipulation group had a significantly higher improvement rate than the other three groups (*P* < 0.05). No significant differences were found among the manipulation, acupuncture, and traction groups (*P* > 0.05). The acupuncture group showed significantly better improvement than the traction group (*P* < 0.05). At the 3-month follow-up, the acupuncture combined with manipulation group continued to outperform the manipulation and traction groups, with statistical significance (*P* < 0.05). No significant differences were observed among the manipulation, acupuncture, and traction groups (*P* > 0.05). The acupuncture group again showed significantly better improvement than the traction group (*P* < 0.05). Detailed results are provided in Table 4 and Figure 5.

**TABLE 4:** Clinical efficacy assessment across study time points.

Time	AM group (*n* = 55)	Ac group (*n* = 52)	SM group (*n* = 52)	Tr group (*n* = 51)
**1 week**				
Excellent, *N* (%)	1 (1.67%)	0 (0.00%)	0 (0.00%)	0 (0.00%)
Good, *N* (%)	6 (10.00%)	0 (0.00%)	0 (0.00%)	0 (0.00%)
Fair, *N* (%)	32 (53.33%)	18 (30%)	20 (33.33%)	6 (10.00%)
Poor, *N* (%)	21 (35.00%)	42 (70%)	40 (66.67%)	54 (90.00%)
**3 week**				
Excellent, *N* (%)	3 (5.00%)	0 (0.00%)	1 (1.67%)	0 (0.00%)
Good, *N* (%)	35 (58.33%)	26 (43.33%)	25 (41.67%)	12 (20.00)
Fair, *N* (%)	20 (33.33%)	27 (45.00%)	22 (36.67%)	28 (46.67%)
Poor, *N* (%)	2 (3.33%)	7 (11.67%)	12 (20.00%)	20 (33.33%)
**1 month follow-up**				
Excellent, *N* (%)	12 (21.05%)	2 (3.70%)	7 (13.21%)	1 (1.89%)
Good, *N* (%)	36 (63.16%)	30 (55.56%)	22 (41.51%)	20 (37.74%)
Fair, *N* (%)	7 (12.28%)	16 (29.63%)	20 (37.74%)	16 (30.19%)
Poor, *N* (%)	2 (3.51%)	6 (11.11%)	4 (7.55%)	16 (30.19%)
**3 month follow-up**				
Excellent, *N* (%)	8 (14.55%)	3 (5.77%)	1 (1.92%)	1 (1.96%)
Good, *N* (%)	36 (65.45%)	29 (55.77%)	26 (50.00%)	19 (37.25%)
Fair, *N* (%)	8 (14.55%)	17 (32.69%)	17 (32.69%)	12 (23.53%)
Poor, *N* (%)	3 (5.45%)	3 (5.77%)	8 (15.38%)	19 (37.25%)

### 3.5 Safety assessment

Throughout the trial, a total of 18 participants experienced adverse reactions. In the acupuncture combined with spinal manipulation group, two participants experienced palpitations, dizziness, and cold sweats related to needle shock. These symptoms were alleviated by having the participants lie flat and drink warm water, with symptoms improving within 10 min, and no further adverse reactions were noted. In the acupuncture combined with manipulation group, three participants, in the manipulation group, five participants, and in the traction group, four participants experienced worsening low back and leg pain after the initial treatment. For those whose symptoms worsened after manipulation, gentle local manipulation was provided, and the intensity and duration of the manipulation were adjusted after communication with the patients. For traction group participants, the traction weight was reduced, or the duration of traction was shortened as appropriate. The overall incidence of adverse events was 8.57%, with 9.09% in the acupuncture combined with spinal manipulation group, 7.69% in the acupuncture group, 9.62% in the manipulation group, and 7.84% in the traction group. There were no statistically significant differences among the groups (*P* > 0.05).

## 4 Discussion

In this study, we systematically compared traditional Chinese spinal manipulation combined with acupuncture, manual therapy, acupuncture alone, and traction therapy for the first time. Consistent with Saal and Saal’s (19) study, approximately 90% of LDH patients achieved good or excellent outcomes with conservative treatment. All four non-pharmacological therapies demonstrated favorable therapeutic effects. During the follow-up (1 month and 3 months post-treatment), VAS and JOA scores consistently remained better than pre-treatment levels, with no rebound, indicating that the short-term efficacy of non-pharmacological treatments for LDH is satisfactory. Furthermore, our study revealed a synergistic effect between manipulation (spinal manipulation) and acupuncture. Patients with LDH who received a combination of acupuncture and spinal manipulation showed significantly greater improvement in VAS and JOA scores during treatment and follow-up compared to the other three groups. Additionally, their clinical efficacy was notably superior to the other three groups. Although there was no significant difference between manipulation and acupuncture overall, subgroup analysis of JOA scores revealed that, at 3 weeks of treatment, the acupuncture group had significantly better lower limb numbness scores (1.44 [1.32, 1.52]) and pain scores (1.71 [1.43, 1.85]) compared to the manipulation group (1.41 [1.23, 1.51], 1.69 [1.45, 1.88]) and traction group (1.38 [1.21, 1.44], 1.55 [1.42, 1.69]). However, the manipulation group showed significantly greater improvement in low back pain (1.57 [1.31, 1.65]) and lumbar flexion activity (1.41 [1.23, 1.59]) compared to the acupuncture group (1.56 [1.32, 1.27], 1.31 [1.20, 1.59]). Additionally, the traction group showed better improvement in lumbar forward bending (1.40 [1.12, 1.49]) compared to the acupuncture group (1.31 [1.20, 1.59]). At the 1-month and 3-month follow-ups, there were no significant statistical differences between the acupuncture and manipulation groups across JOA subgroups. However, both groups performed better than the traction group in terms of low back pain, washing, and sitting scores.

Lumbar disc herniation primarily involves the bladder meridian, Du meridian, and gallbladder meridian within the body’s energy pathways (20). The Ya-jiaji acupoint is the preferred point for treating LDH, as it is located between the Du meridian and the bladder meridian, playing a critical role in coordinating the two. Acupuncture at Ya-jiaji promotes blood circulation in the lumbar region and beneath the spinous processes (21). Moreover, Ya-jiaji is intricately connected to the anterior and posterior roots of the spinal nerves. Acupuncture at Ya-jiaji can regulate sensory and motor functions throughout the body and its internal organs (22). However, acupuncture point selection often focuses on local effects (“where the acupoint is located, it treats”), primarily addressing local pathological changes, while neglecting the holistic principle of “where the meridian passes, it treats.” Therefore, in this study, we based our approach on the theories of “drawing yin from yang and yang from yin” and “seeking harmony in qi.” We applied acupuncture to the contralateral limb of the affected area, utilizing the physiological characteristics of the meridians that connect the upper and lower parts, as well as the left and right sides of the body, to harmonize yin and yang, smooth the meridians, and alleviate pain.

Acupuncture has been shown to effectively alleviate sciatica caused by LDH (23–25). However, Yuan et al. (26) questioned the efficacy of acupuncture in improving lumbar functional mobility. Our study also indicated that the spinal manipulation group exhibited significantly greater improvement in low back pain and lumbar flexion mobility in LDH patients compared to the acupuncture group. Additionally, at week 3 of treatment, traction demonstrated superior efficacy over acupuncture in improving lumbar forward flexion mobility. Although acupuncture can alleviate symptoms in LDH patients by promoting relaxation of tense, spasmodic muscles, it may have limitations as a standalone treatment. This is primarily because acupuncture can only gradually alter minor joint dysfunctions by alleviating localized muscle spasms and reducing stress concentration effects. However, it is less effective in repositioning already displaced facet joints and improving the intraspinal environment, potentially leading to a slower improvement in localized lumbar function (27).

Spinal manipulation focuses on muscle relaxation, knot release, and realignment (28). The mechanical properties of the intervertebral disc are significant in patients with LDH due to loss of disc water, matrix decomposition, and fibrous annulus cleft formation (29). Spinal manipulation improves the intervertebral space height and restores the mechanical balance of the vertebral body through traction, repositioning and release maneuvers. Muscle relaxation therapy involves releasing local muscles by applying pressure to the gluteal muscles with one hand while the other hand relaxes and manipulations the sacrospinalis from top to bottom, facilitating the relaxation of lumbar muscles, especially the iliopsoas and erector spinae in the lumbosacral region (16). Knot release therapy targets nodules, cord-like structures, and tender points formed in diseased local muscles. Pain often originates at attachment points of muscles, ligaments, fascia, and along the course of cutaneous or sciatic nerves (30). Techniques like pinpointing, lifting, pressing, and rolling are used on these points to relieve soft tissue adhesions, stimulate blood circulation, and relax muscles. When using the lateral oblique manipulation, if the lesion is in the upper lumbar vertebrae, the lower body should be twisted more than the upper body. Conversely, if the lesion is in the lower lumbar vertebrae, the upper body should be twisted more than the lower body. This approach helps to correct the disordered spatial sequence of lumbar facet joints and vertebral load-bearing lines (31), improving the effective space of intervertebral foramina and non-continuous bony spinal canals, reducing or eliminating mechanical compression and irritation of nerves and blood vessels, and restoring dynamic stress balance in the lumbar spine (32). Finally, the prone traction and shaking method is used to relieve lumbar muscle spasms, widen intervertebral spaces, and reduce nerve compression. Additionally, performing pelvic rotation, supine bridging, and lifting the lower limbs under supine pelvic traction can promote the retraction of protruded nucleus pulposus. Thus, the combined use of spinal manipulation and acupuncture can help the body achieve a balanced state of bone realignment, muscle relaxation, meridian flow, and yin-yang harmony.

Therefore, the combination of acupuncture and spinal manipulation may offer potential benefits in restoring lumbar spine function and stability while alleviating pain. Our study results also indicate that the group receiving combined acupuncture and spinal manipulation showed significantly better relief of low back and leg pain, as well as nerve function recovery, in patients with LDH compared to the other three groups. Additionally, the duration and maintenance of symptom relief in LDH were particularly prominent, possibly due to spinal manipulation correcting abnormal lumbar structures, restoring vertebral stability, relieving lumbar muscle spasms, and promoting the flow and nourishment of qi and blood. This, combined with acupuncture, achieves the effects of regulating the Du and bladder meridians, promoting qi and blood circulation, relaxing muscles, and relieving pain.

Although this study provides valuable insights into the efficacy of non-pharmacological treatments for LDH, certain limitations warrant consideration. Firstly, the study size and follow-up period may not fully reflect the long-term impact of this treatment on patient prognosis. Expanding the participant pool and extending follow-up time will provide a more comprehensive perspective. Secondly, due to the specificity of the treatment measures, it was impossible to blind physicians and patients, although we made efforts to blind researchers responsible for data collection, final evaluations, and statistical analysis, subjective biases in results are unavoidable. Thirdly, our outcome measures were all subjective, lacking support from objective indicators such as lumbar MRI and X-ray imaging. Thus, our next research step will further elucidate the differences between imaging changes and clinical efficacy in LDH patients, exploring their biomechanical relationships.

## 5 Conclusion

According to the study results, non-pharmacological therapies show good short-term efficacy in improving clinical symptoms and reducing leg pain in LDH patients. Furthermore, the combination of acupuncture and spinal manipulation has additive effects in LDH treatment, efficiently alleviating lumbar and leg pain while restoring lumbar spine function, demonstrating practical value and utility. Therefore, we strongly recommend incorporating combined acupuncture and spinal manipulation therapy into LDH treatment protocols. This approach significantly improves LDH symptoms while prioritizing patient safety, thus facilitating comprehensive recovery. We suggest future research delve deeper into non-pharmacological therapies, including long-term follow-up studies, biomechanical analyses, and exploration of underlying mechanisms.

## Data Availability

The original contributions presented in this study are included in this article/supplementary material, further inquiries can be directed to the corresponding author.
